# Frequency and associated factors of accommodative and non-strabismic binocular vision dysfunctions among clinical adults in Western China: A cross-sectional study

**DOI:** 10.1097/MD.0000000000043881

**Published:** 2025-08-22

**Authors:** Dilimulati Yushan, Zhang Hong

**Affiliations:** a Department of Ophthalmology, Eye Institute, First Affiliated Hospital of Xinjiang Medical University, Urumqi, China.

**Keywords:** accommodative insufficiency, convergence insufficiency, non-strabismic binocular visual dysfunction, optometry clinic

## Abstract

This study aims to determine the prevalence and identify associated risk factors of accommodative and non-strabismus binocular vision dysfunctions among clinical adults aged 30 to 44 years in Western China. Consecutive eligible outpatients who visited the optometry clinic at the First Affiliated Hospital of Xinjiang Medical University were recruited. Comprehensive assessments of accommodative and binocular vision were conducted, including subjective and objective refractive status examination, Worth 4 dot test, distant and near horizontal eye position (Von Graefe method), positive fusional vergence (distance and near), negative fusional vergence (distance and near), near point of convergence (NPC), accommodative response (BCC, binocular cross-cylinder test), negative relative accommodation (NRA), positive relative accommodation, monocular accommodation amplitude (MAA; negative lenses), monocular accommodative facility (MAF; ±2.00 D flip mirror), and vergence facility (VF; 3ΔBI/12ΔBO). Prior to the start of the above examinations, a questionnaire survey was conducted using the convergence insufficiency symptom survey (CISS) designed by convergence insufficiency treatment trial (CITT). The results indicated that 64.83% of participants exhibited accommodative and non-strabismic binocular vision dysfunctions, of which 42.07% were classified as accommodative dysfunction and 37.25% as binocular anomalies. Among these dysfunctions, accommodative insufficiency was the most prevalent, accounting for 37.25%, followed by convergence insufficiency (CI) at 24.83% and convergence excess at 6.21%. A significant linear correlation was observed between the spherical equivalent (SE) of the right eye and parameters including convergence function, MAA, MAF, and positive fusion images at far range. Additionally, the CISS score was found to be significantly associated with the distance horizontal strabismic, stereopsis, NPC, MAA and MAF. Furthermore, the hours of near vision tasks were correlated with the distant positive and negative fusion ability, NPC and MAA. Multinomial logistic regression was conducted to identify potential risk factors associated with accommodative and non-strabismic binocular vision dysfunction. The results showed that hours of doing near vision tasks ≥ 6 hours and binocular SE difference ≥ 1.5 D were relevant factors for the frequency of accommodative and non-strabismic binocular vision abnormalities. Approximately two-thirds of the optometric clinical population in Western China – including individuals in the pre-presbyopic stage – may be affected by accommodative or non-strabismic binocular vision dysfunctions, potentially linked to prolonged near vision tasks.

## 1. Introduction

Accommodative and non-strabismic binocular vision dysfunctions include isolated accommodative dysfunctions, non-strabismic binocular vision disorders, or a combination of both.^[1]^ They are a range of visual impairments that affect subjects’ binocular vision and visual performance during performing near vision tasks. Accommodative dysfunctions are subcategorized into accommodative insufficiency (AI), accommodative excess, and accommodative infacility (AIF).^[2]^

Non-strabismic binocular vision disorders are classified as convergence insufficiency (CI), convergence excess, divergence insufficiency, divergence excess, fusional vergence dysfunction, basic exophoria (BX) and basic esophoria.^[2]^ These dysfunctions often result in symptoms such as eye strain, headaches, blurred vision, and diplopia, affecting the clarity, comfort, and efficiency of binocular vision during near vision tasks such as reading, writing, and computer work.^[3,4]^

Symptoms and prevalence values of accommodative and non-strabismic binocular vision dysfunctions were reported inconsistently in different kinds of studies.^[5–10]^ Many factors such as study populations,^[11]^ examination methods and the various diagnostic criteria^[9]^ may contribute to this difference. Although research on accommodative and non-strabismic binocular vision dysfunctions is well-established internationally, there remains a lack of comprehensive epidemiological data regarding the prevalence of these conditions among adults aged 30 to 44 years in China. This age range encompasses adults who are in the pre-presbyopia stage (35–44 years).^[12]^ Individuals in this age group are more prone to ocular symptoms due to a decline in accommodative ability and accommodative convergence function caused by natural aging. However, these symptoms are often overlooked in clinical practice, leading to delayed diagnosis and treatment, which can cause significant physical and psychological distress to patients. Moreover, these conditions may worsen or evolve into strabismic dysfunction, potentially resulting in stereoscopic vision loss and visual suppression.^[13,14]^

The primary objective of this study is to estimate the prevalence of these abnormalities among outpatients aged 30 to 44 years attending the optometry clinic at the First Affiliated Hospital of Xinjiang Medical University, as well as to identify associated risk factors. This investigation aims to enhance understanding of the most common types of binocular vision disorders and related symptoms in adult patients, thereby assisting clinicians in improving early recognition and diagnosis.

## 2. Participants and methods

This study was approved by the Ethics Committee of Xinjiang Medical University (Approval No. K202407-09) and conducted in accordance with the principles of the Declaration of Helsinki. All the subjects signed an informed consent form.

### 2.1. Participants

This study enrolled 145 adult outpatients aged 30 to 44 years who visited the optometry clinic at the First Affiliated Hospital of Xinjiang Medical University, all of whom had a best-corrected visual acuity of at least 0.8 in both eyes. The following exclusion criteria were applied: any ocular organic disease affecting visual acuity or binocular visual function; a history of amblyopia and tropia; prior ocular surgery, including refractive or strabismus surgery; extraocular muscle injuries affecting ocular motility for any reason; and any use of medications that may affect visual acuity or binocular visual function within the past 3 months.

### 2.2. Testing procedures

1. All participants underwent a comprehensive ophthalmic evaluation, including slit-lamp biomicroscopy of the anterior segment, fundus examination, and assessment of binocular visual function. Prior to undergoing comprehensive binocular vision assessment, all participants completed the convergence insufficiency symptom survey (CISS) questionnaire, which evaluated the frequency and severity of common symptoms associated with accommodative and binocular vision dysfunctions. Responses were rated on a scale of “never,” “rarely,” “sometimes,” “frequently,” or “always,” with each item scored from 0 to 4, resulting in a total possible score ranging from 0 to 60. A symptom was considered present if he/she reported experiencing it at least “sometimes” (scores ≥ 2). Participants with accommodative and binocular vision disorders were further categorized into symptomatic and asymptomatic groups based on the CISS scores, with scores of 21 or higher on the 15 symptom items classified as symptomatic.^[15]^
2. Objective and subjective assessments of refractive error (RE) is to obtain the best visual acuity for all subjects. The RE results were converted to sphere equivalent (SE) for statistical analysis of the data. Cycloplegic refraction was contraindicated in all subjects in this study because of the need to assess accommodative and binocular vision functions.
3. Binocular visual assessment was performed on the basis of best visual acuity in the following order: stereo vision was measured with the Titmus stereo test at a distance of 40 cm. The Worth 4 dot test was conducted at both distance and near fixation as part of the sensory function assessment. Horizontal phorias, along with positive and negative fusional vergence capacities, were evaluated using the von Graefe method (Topcon CV-5000 automated vision tester) under both distance and near visual conditions, record the average of 3 repeated measurements as the final result. The negative fusional vergence was measured before the positive fusional vergence. A flipper with a combination of 3^∆^ base in and 12^∆^ base out was used to assess vergence facility at 40 cm, and record the number of cycles completed in 1 minutes (completing the flip of 2 prisms counts as 1 cycle, cpm). The near point of convergence (NPC) was measured with an accommodative target of automated vision tester at a distance of 40 cm. Negative and positive relative accommodations were tested at a distance of 40 cm. Accommodative responses were measured using ± 0.50 DC binocular cross-cylinder mirrors. Monocular accommodation amplitude (MAA) was determined by the minus lens method. To assess monocular accommodative facility (MAF), participants were required to focus on a 20/30 accommodative visual target card at a distance of 40 cm. The right eye is measured first, followed by the left eye, using a flipper that combines + 2.00 DS and −2.00 DS lenses for testing. The number of flip repetitions was recorded over a 60-second period and subsequently converted into cycles per minute (cpm).
4. Myopia and hyperopia were defined respectively as spherical degree ≤ −0.50 D and spherical degree ≥+0.75 D. Astigmatism was defined cylindrical lens degree > 0.50 D. Presbyopia was defined as the inability to read N8 (equivalent to 6/12) binocularly at a reading distance of 40 cm or at the individual’s habitual working distance in individuals aged ≥ 35 years^[16]^ (calculation method). Accommodative and binocular vision assessments were conducted with the participants’ binocular refractive correction. The classification criteria for accommodative and non-strabismic binocular vision dysfunctions were primarily derived from the work of Scheiman and Wick,^[17]^ whereas the diagnostic criteria were based on the clinical guidelines proposed by Hussaindeen et al.^[2]^

### 2.3. Statistical analysis

The sample size was calculated using Power Analysis and Sample Size 15 software based on the research by Lara et al,^[5]^ which determined a minimum sample size of 130 cases. Statistical analysis was performed using SPSS 27.0. For monocular measurements, data from the right eye were used. Categorical data were expressed as frequencies and percentages. Pearson’s or Spearman’s correlation coefficients were calculated for correlation analysis, depending on the normality of data distribution. Multivariable logistic regression analysis was performed to identify potential risk factors associated with accommodative and non-strabismic binocular vision dysfunctions. All statistical tests were 2-tailed, and a *P*-value < .05 was considered statistically significant.

## 3. Results

**Figure 1.**
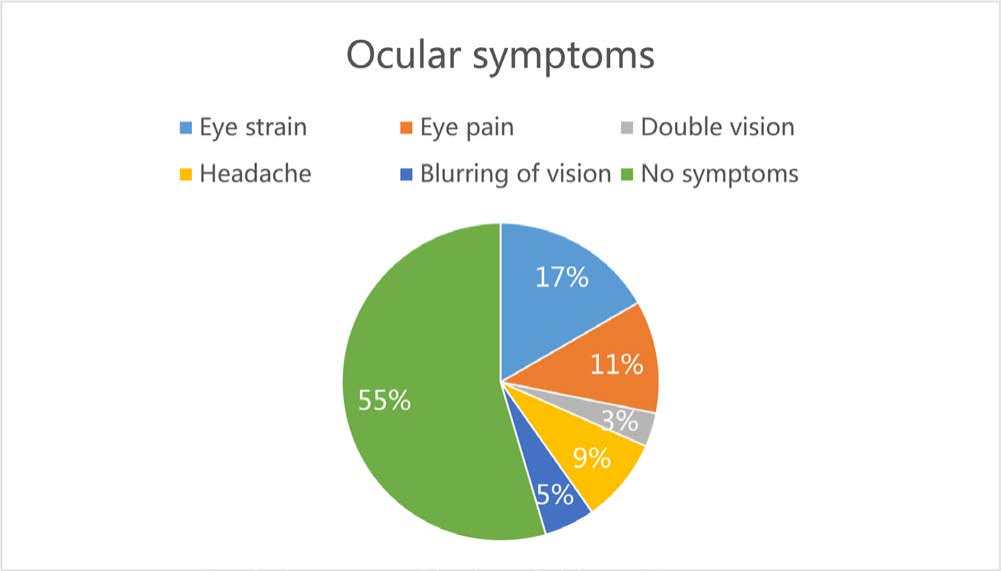
Ocular symptoms of outpatients in this study:the most common binocular vision symptom was eyestrain (17.0%), followed by eye pain (11%).

From November 2023 to May 2024, a total of 145 outpatients aged 30 to 44 years were recruited from the optometry clinic of the First Affiliated Hospital of Xinjiang Medical University for a routine vision examination, with a mean age of 36.4 ± 5.0 years, and 55 (37.93%) were male. 118 (81.34%) participants had RE, with an average spherical equivalent (SE) of −2.8 ± 2.91 D.96 (66.21%) of them were myopic with a refractive range of (−0.50 to −12.50 D), 7 (4.8%) were hyperopic and 80 (55.20%) were astigmatic. Only 8 (8.28%) participants’ binocular SE difference ≥ 1.5 D.82 (56.55%) participants were in the pre-presbyopia stage, and 36 (24.83%) were diagnosable as presbyopia. 50 (34. 48%) were symptomatic (CISS scores ≥ 21) and 95 (65.52%) were asymptomatic (CISS scores < 21), and symptomatic group was found higher among participants with accommodative and non-strabismic binocular vision dysfunction (*χ*^2^ = 7.71, *P* < .05), as shown in Table 1. The most prevalent symptom was eyestrain (17.0%), followed by eye pain (11%) (Fig. 1).

**Table 1:** Number of participants with symptomatic versus asymptomatic accommodative and non-strabismic binocular vision dysfunction.

Parameters	Accommodative and non-strabismic binocular vision dysfunction	Normal BV	Total
Symptomatic	40 (80.0%)	10 (20.0%)	50
Asymptomatic	54 (56.84%)	41 (43.16%)	95
* *χ^2^		7.71	
* P*		.006	

Of the 145 outpatients, 94 were diagnosed with accommodative and non-strabismic binocular vision dysfunction, comprising 64.83% (38 male and 56 female).^[18]^ Of all the participants, 42.07% had accommodative disorders and 37.25% had a binocular vision dysfunction. The most common type of accommodative disorder was AI, with an overall prevalence of 37.25% (54/145). CI was the most common type of binocular vision dysfunction, with an overall prevalence of 24.83% (36/145). Table 2 illustrates the comparison of the frequency of accommodative and non-strabismic binocular vision dysfunction between this study and other studies. Table 3 shows the prevalence of accommodative and non-strabismic binocular vision dysfunction at different age groups, gender and refractive status.

**Table 2:** Comparisons of frequency between other studies and the present study on accommodative and non-strabismic binocular vision dysfunction.

Study	Lara et al^[5]^	Garcia-Munoz et al^[6]^	Hoseini-Yazdi et al^[7]^	Darko-Takyi et al^[8]^	Franco et al^[9]^	Liu et al^[10]^	Current study
Country	Spain	Spain	Iran	Ghana	Portugal	China	China
Sample size	265	175	83	105	156	172	145
Age	10–35	18–35	21.3 ± 3.5	19–27	18–35	12–35	30–44
AI (%)	3.0	–	2.4	4.70	11.54	0.6	37.24
AIF (%)	–	–	–	6.70	5.77	4.0	3.45
AE (%)	6.4	2.29	3.6	1.90	3.85	4.6	1.38
CI (%)	0.8	3.43	3.6	1.90	7.05	12.8	24.83
BX (%)	0.4	0.57	1.2	1.90	0	2.9	0
BE (%)	–	1.14	–	4.70	0	1.2	0
CE (%)	4.5	2.29	2.4	1.00	3.85	6.4	6.21
DE (%)	–	0.57	–	–	0	0.6	0
DI (%)	–	–	–	2.90	0	0	2.07
CI + AI (%)	0.4	1.14	–	–	–	0.6	8.28
CI + AE (%)	1.9	0.57	1.2	–	–	–	0.69
CE + AE (%)	2.6	–	1.2	–	–	–	0
CE + AI (%)	1.9	0.57	1.2	–	–	–	2.07
CI + AIF (%)	0.4	–	–	–	–	0.6	0.69
AE + AIF (%)	–	–	1.2	–	–	–	0
BX + AE (%)	–	–	1.2	–	–	0.6	0
FVD (%)	–	–	–	–	0	2.9	4.14
FVD + AI	–	0.57	–	–	–	–	0.69
RE (%)	–	45.14	–	59.0	66.7	96.5	81.34
Total (%)	22.3	13.1	19.3	34.30	32.05	36.05	64.83

AE = accommodative excess, AI = accommodative insufficiency, AIF = accommodative infacility, BE = basic esophoria, BX = basic exophoria, CE = convergence excess, CI = convergence insufficiency, DE = divergence excess, DI = divergence insufficiency, FVD = fusional vergence dysfunction.

**Table 3:** Number of participants with accommodative and non-strabismic binocular vision dysfunction at different age groups, gender and refractive status.

Parameters	Accommodative and non-strabismic binocular vision dysfunction	Normal BV	Total
Age groups			
30–35 yr	47	25	72 (49.66%)
36–40 yr	22	14	36 (24.83%)
41–44 yr	25	12	37 (25.52%)
Gender			
Male	38	17	55 (37.93%)
Female	56	34	90 (62.07%)
Refractive status			
Myopia	66	30	96 (66.21%)
Hyperopia	5	2	7 (4.83%)
Astigmatism	54	26	80 (55.20%)

**Figure 2.**
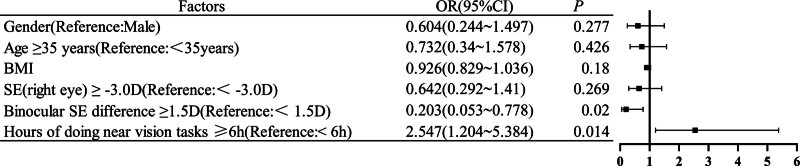
Logistic regression analysis of associated factors of accommodative and non-strabismic binocular vision dysfunction:near eye time ≥ 6h (*P* = .014 < .05) and binocular SE difference ≥ 1.5 D (*P* = .02 < .05) were statistically significant.

In this study, a significant linear correlation was observed between the SE of the right eye and parameters including convergence function, MAA, MAF, and positive fusion images at far range. The greater the SE of the right eye, the higher the index of these binocular vision indices except NPC (Table 4). Additionally, the CISS score was found to be significantly associated with the distance horizontal strabismic, stereopsis, NPC, MAA and MAF (Table 5). Furthermore, the hours of near vision tasks were correlated with the distant positive and negative fusion ability, NPC and MAA (Table 6). Lastly, multiple logistic regression was used to analyze the influencing factors of accommodative and non-strabismic binocular vision dysfunction. The results showed that hours of doing near vision tasks ≥ 6 hours and binocular SE difference ≥ 1.5 D were significantly associated with the prevalence of accommodative and non-strabismic binocular vision abnormalities (Fig. 2).

**Table 4:** Correlation between refractive status and binocular vision measures.

Iteams	Binocular SE difference		SE of right eye	
	*r*	*P*	*r*	*P*
Stereopsis	0.114	.172	0.006	.940
Distance horizontal heterophoria	−0.065	.438	−0.004	.962
ear horizontal heterophoria	0.034	.684	−0.097	.247
Near BO break point	−0.058	.487	−0.265	.001*
Near BI break point	0.084	.314	−0.165	.048*
Distance BO break point	−0.052	.537	−0.243	.003*
Distance BI break point	−0.043	.606	−0.085	.309
Near VF, cpm	−0.068	.419	−0.160	.054
NPC, cm	0.090	.284	0.249	.003*
MAA, D	−0.030	.722	−0.175	.035*
MAF, cpm	–	–	−0.242	.003*
CISS	−0.023	.786	0.122	.143

n = 145.

BI = base in, BO = base out, CISS = convergence insufficiency symptom survey, MAA = monocular accommodation amplitude, MAF = monocular accommodative facility, NPC = near point of convergence, SE = spherical equivalent, VF = vergence facility.

* The correlation was statistically significant, *P* < .05.

**Table 5:** Correlation between CISS score and binocular vision measures.

Iteams	CISS	
	*r*	*P*
Stereopsis	0.249	.002*
Distance horizontal heterophoria	−0.020	.816
Near horizontal heterophoria	−0.183	.028*
Near BO break point	−0.013	.874
Near BI break point	−0.058	.486
Distance BO break point	−0.015	.857
Distance BI break point	0.024	.773
Near VF, cpm	−0.058	.487
NPC, cm	0.281	.001*
MAA, D	−0.411	.000*
MAF, cpm	−0.358	.000*

n = 145.

BI = base in, BO = base out, MAA = monocular accommodation amplitude, MAF = monocular accommodative facility, NPC = near point of convergence, VF = vergence facility.

* The correlation was statistically significant, *P* < .05.

**Table 6:** Correlations between hours of near vision tasks and various binocular vision indicators.

Iteams	Hours of near vision tasks	
	*r*	*P*
Stereopsis	−0.029	.002
Distance horizontal heterophoria	−0.036	.816
Near horizontal heterophoria	0.053	.028
Near BO break point	−0.063	.874
Near BI break point	0.037	.486
Distance BO break point	−0.185	.027*
Distance BI break point	0.264	.001*
Near VF, cpm	0.016	.85
NPC, cm	0.221	.006*
MAA, D	−0.402	.000*
MAF, cpm	−0.096	.135

n = 145.

BI = base in, BO = base out, MAA = monocular accommodation amplitude, MAF = monocular accommodative facility, NPC = near point of convergence, VF = vergence facility.

* The correlation was statistically significant, *P* < .05.

## 4. Discussion

Accommodative and non-strabismic binocular vision dysfunctions are frequently encountered in routine optometric practice.^[5–10]^ However, the frequency varies among different age groups. Recent studies have documented a high prevalence of binocular vision dysfunction among university students, with reported rates ranging from 32.3% to 42%.^[19]^ The study by Tiwari et al^[20]^ demonstrated a 50% frequency of accommodative and non-strabismic binocular vision dysfunction in the 31 to 40 years age group. We found that about two-thirds of the participants exhibited accommodative and non-strabismic binocular vision dysfunction, which is higher than studies mentioned above. These variations may be largely attributable to the source of the study populations – namely, whether they were clinical or non-clinical cohorts. This discrepancy could be explained by the fact that individuals with visual impairments or heightened visual awareness are more likely to seek professional eye care. Additionally, differences in daily near-work duration may also contribute to the observed prevalence rates.

In the present study, we found that participants who spent over 6 hours at near vision tasks were more likely to develop accommodative and non-strabismic binocular vision dysfunction than those who spent less than 6 hours. Porcar et al^[21]^ found that among the 89 video display units users, accommodative and non-strabismic binocular dysfunctions patients accounted for 22.5%, with convergence excess being the most common. In a study by Chiranjib et al, 40% of students at a Malaysian private university who used visual display units were found to exhibit non-strabismic binocular vision anomalies. Among VDU users, the prevalence rates of accommodative and vergence anomalies were reported as 17.86% and 22.14%, respectively. In contrast to our findings, no significant association was observed between daily VDU usage duration and the occurrence of non-strabismic binocular vision anomalies.^[22]^ The observed differences in outcomes between the 2 studies may be attributed to variations in age distribution, sample size, diagnostic criteria, and ethnic background.

Tiwari et al^[20]^ conducted a similar study held in India among MBBS and nursing students, found prevalence of accommodative and non-strabismic binocular vision dysfunction as 41.17% in < 20 years age group, 40.29% in 21 to 25 years age group and 66.66% between 26 to 30 years age group. This also shows higher prevalence of accommodative and non-strabismic binocular vision dysfunction among older age group. However, these prevalence rates are still lower than our study. In this study, we speculated this difference in prevalence may be attributed to different age ranges and diverse sample populations. Ultimately, our findings indicated that age was not a significant factor associated with the prevalence of accommodative and non-strabismic binocular vision dysfunctions (Fig. 2) and no statistical significance between different age groups (χ^*2*^ = 12.66, *P* > .05) (Table 7).

**Table 7:** Distribution of outpatients among different age groups with/without accommodative and non-strabismic binocular vision dysfunction.

Parameters	Accommodative and non-strabismic binocular vision dysfunction	Normal BV	Total
30–35 yr	47	25	72
36–40 yr	22	14	36
41–44 yr	25	12	37
* *χ^2^		12.66	
* P*		.841	

Normal BV = normal binocular vision.

In the present study, 42.07% of outpatients presented an accommodative disorder being the AI the most prevalent (37.24%), followed by AIF (3.45%). And CI (24.83%) is the most common binocular vision dysfunction. Consistent with previous studies, we observed a higher prevalence of accommodative dysfunctions compared to binocular vision dysfunctions.^[6,23]^ Besides, the prevalence of accommodative disorders observed in our study was higher than that reported in previous studies,^[2–7]^ including the findings by Montés-Micó^[24]^ in a clinical population of Valencia, Spain. He reported that 34.6% of participants exhibited at least 1 type of accommodative dysfunction, with AI being the most prevalent subtype (11.4%), followed by accommodative infacility (10.3%). However studies conducted on Spanish college students,^[6]^ Nepalese medical students,^[25]^ urban and rural populations in Tamil Nadu, India,^[2]^ students in Addis Ababa, Nigeria,^[26]^ and optometry clinics in Northeast Sichuan^[10]^ have all reported a higher prevalence of binocular vision disorders, with CI being the most prevalent. Still, the prevalence of CI (24.83%) in our study was higher than all the above studies.

With regard to accommodative disorders, Wajuihian^[27]^ reported that accommodative dysfunction was the most prevalent condition among Black South African individuals aged 10 to 40 years. Lara et al^[5]^ examined a clinical cohort of 265 symptomatic individuals aged 10 to 35 years and reported a prevalence of 9.4% for accommodative dysfunctions. In the study by Liu et al,^[10]^ the prevalence rates of overall accommodative dysfunctions and AI were reported as 9.20% and 0.6%, respectively. However, these prevalence rates were significantly lower than those observed in our study. In turn, Franco et al^[9]^ discovered that accommodative dysfunctions found was 21.16%, and AI at 11.5% was the most frequent functional abnormality among the Portuguese optometry clinic population. Although this value are higher than that obtained by Liu et al^[10]^ it is still considerably lower than that observed in our study. Both studies share the commonality of having study populations under 35 years old and originating from optometric clinics. However, the 2 studies mentioned above, and our study differed from each other in their categorization and diagnostic criteria. Furthermore, the age group of our study population was higher than the age group of the other 2 study populations.

The age-related decline in accommodative amplitude is primarily attributed to reduced viscoelastic properties and structural alterations in the crystalline lens, which are well-established physiological changes associated with aging.^[28,29]^ The amplitude of accommodation represents a critical parameter in the diagnosis of accommodative dysfunctions, particularly as the methodology employed for its assessment may significantly influence the resulting measurements. In our study, among the 82 pre-presbyopic outpatients aged 35 to 44 years, 29.27% (24/82) had both accommodative and non-strabismic binocular vision dysfunction and presbyopia, 32.93% (27/82) had only accommodative and non-strabismic binocular vision dysfunction and 23.17% (19/82) had neither dysfunction. There was no statistical significance between with presbyopia and without presbyopia groups (χ^2^ = 13.61, *P* > .05) (Table 8). Moreover, we observed significant reductions in monocular accommodation amplitude (MAA) with increasing of presbyopia degree. According to study of Burns et al,^[30]^ 5 different methods may be used: pushup, push-down, push-down to recognition, negative lenses (Sheard’s method) and dynamic retinoscopy. We used negative lens method in this study. However, Taub et al^[31]^ reported that the negative lens method yields lower measurements of accommodative amplitude compared to other techniques. The pushup method, in contrast, has been shown to produce the most consistent results in adults when referenced against normative data. This discrepancy in methodology may partially explain the higher prevalence of accommodative dysfunctions observed in our study. Furthermore, it is likely that many similar studies have restricted their inclusion criteria to individuals aged 35 years or younger, in an effort to exclude accommodative anomalies associated with age-related presbyopic changes.

**Table 8:** Distribution of accommodative and non-strabismic binocular vision dysfunction among outpatients aged 35 to 44 yr with/without presbyopia.

Parameters	Accommodative and non-strabismic binocular vision dysfunction	Normal BV	Total
With presbyopia	24	12	36
Without presbyopia	27	19	46
* *χ^2^		13.61	
* P*		.460	

Normal BV = normal binocular vision.

For binocular vision dysfunction, 37.25% of outpatients with or without symptom presented a non-strabismic binocular vision dysfunction being the CI the most prevalent (24.83%), of which 15.17% (22/145) were diagnosed CI with ocular symptoms. Both prevalence rates of CI in our study are higher than the prevalence rates of CI in Franco et al^[9]^ (6.9%) and Liu et al^[10]^ (12.8%) studies. They also used the von Grafe technique for measure the horizontal phoria and their participants are from from a clinic population. Gantz and Stiebel-Kalish discovered that the high prevalence of myopia (93.0%) may contribute to the elevated incidence of CI, which is one of the most common binocular vision dysfunctions and affects approximately 7.5% of the general population.^[32]^ Age-related differences between NPC (break) and NPC (recovery) in presbyopic individuals may represent another contributing factor to the high incidence of CI. In a population of 2433 individuals aged 10 to 86 years, NPC values were found to increase with age across the entire age range studied, with the most pronounced changes occurring between the 30 to 39 and 40 to 49 years age groups.^[33]^ Spierer and Hefetz followed a cohort of 100 individuals over a 20-year period and observed a progressive increase in NPC as subjects transitioned into the pre-presbyopic stage (34–38 years of age).^[18]^ This may explain the high prevalence of CI observed in this study. Symptoms of CI are typically associated with near-related tasks and may include eye strain, headache, intermittent blurring or diplopia, perceived movement of text on the page, difficulty concentrating, slow reading speed, and loss of place during reading.

In this study, 81.34% (118/145) had a RE, with 66.21% (96/145) were myopic. Most common ocular symptom was eye strain seen in 17% outpatients with accommodative and non-strabismic binocular vision. We observed that higher SE in the right eye was associated with increased values across most binocular vision indices – except for NPC – as shown in Table 4, suggesting a potential relationship between the degree of myopia and the functions of accommodation and convergence. Regression analysis revealed that gender, age, body mass index, and SE of the right eye were not significantly associated with the presence of accommodative and non-strabismic binocular vision. Hours of doing near vision tasks ≥ 6 hours was a risk factor and binocular SE difference ≥ 1.5 D was a protective factor. However, binocular SE difference ≥ 1.5 D was not a relevant factor in the development of accommodative and non-strabismic binocular vision in studies of Liu et al^[10]^ and Cai et al.^[34]^ Woong found that stereoacuity was better in individuals with corrected anisometropia compared to those with uncorrected anisometropia, and that it significantly deteriorated with increasing severity of anisometropia in the uncorrected group.^[35]^ Binocular SE difference ≥ 1.5 D as a protective factor may contradict the findings of several of these studies. Differences may be due to the type of population studied and sample size. Only 8.28% (12/145) of the participants in this study had a binocular SE difference of ≥ 1.5 D, the sample size may be insufficient to adequately capture the complex relationship between binocular SE difference of ≥ 1.5 D and accommodative and non-strabismic binocular vision dysfunction.

There are several limitations to our study. The study population was drawn from a clinical setting and represents a relatively small and geographically limited sample with potential selection bias; therefore, the results may not be fully representative of all adult populations aged 30 to 45 years or directly generalizable to broader populations. Additionally, a more detailed categorical diagnosis was applied in this study, which may have led to an underestimation of some less prevalent types of accommodative and non-strabismic binocular vision dysfunctions.

## 5. Conclusions

Approximately two-thirds of the optometric clinical population in Western China – including individuals in the pre-presbyopic stage – may be affected by accommodative or non-strabismic binocular vision dysfunctions, potentially linked to prolonged near vision tasks.

## Author contributions

**Conceptualization:** Dilimulati Yushan, Zhang Hong.

**Data curation:** Dilimulati Yushan, Zhang Hong.

**Formal analysis:** Zhang Hong.

**Investigation:** Dilimulati Yushan, Zhang Hong.

**Methodology:** Dilimulati Yushan, Zhang Hong.

**Project administration:** Dilimulati Yushan, Zhang Hong.

**Resources:** Dilimulati Yushan, Zhang Hong.

**Software:** Dilimulati Yushan, Zhang Hong.

**Validation:** Zhang Hong.

**Visualization:** Zhang Hong.

**Writing – original draft:** Dilimulati Yushan.

**Writing – review & editing:** Dilimulati Yushan, Zhang Hong.
